# Cocaine Self-Administration and Abstinence Modulate NMDA Receptor Subunits and Active Zone Proteins in the Rat Nucleus Accumbens

**DOI:** 10.3390/molecules25153480

**Published:** 2020-07-31

**Authors:** Irena Smaga, Karolina Wydra, Małgorzata Frankowska, Fabio Fumagalli, Marek Sanak, Małgorzata Filip

**Affiliations:** 1 Maj Institute of Pharmacology Polish Academy of Sciences, Department of Drug Addiction Pharmacology, Smętna 12, PL 31-343 Kraków, Poland; wydra@if-pan.krakow.pl (K.W.); frankow@if-pan.krakow.pl (M.F.); mal.fil@if-pan.krakow.pl (M.F.); 2 Department of Pharmacological and Biomolecular Sciences, Università degli Studi di Milano, Via Balzaretti 9, 20133 Milano, Italy; Fabio.Fumagalli@unimi.it; 3 Department of Internal Medicine, Jagiellonian University Medical College, Skawińska 8, PL 31-066 Kraków, Poland; marek.sanak@uj.edu.pl

**Keywords:** cocaine abstinence, cocaine self-administration, Munc13, NMDA receptor subunit, Rab3A, RIM1

## Abstract

Cocaine-induced plasticity in the glutamatergic transmission and its *N*-methyl-d-aspartate (NMDA) receptors are critically involved in the development of substance use disorder. The presynaptic active zone proteins control structural synaptic plasticity; however, we are still far from understanding the molecular determinants important for cocaine seeking behavior. The aim of this study was to investigate the effect of cocaine self-administration and different conditions of cocaine forced abstinence on the composition of the NMDA receptor subunits and on the levels of active zone proteins, i.e., Ras-related protein 3A (Rab3A), Rab3 interacting molecules 1 (RIM1) and mammalian uncoordinated protein 13 (Munc13) in the rat nucleus accumbens. We found an up-regulation of the accumbal levels of GluN1 and GluN2A following cocaine self-administration that was paralleled by an increase of Munc13 and RIM1 levels. At the same time, we also demonstrated that different conditions of cocaine abstinence abolished changes in NMDA receptor subunits (except for higher GluN1 levels after cocaine abstinence with extinction training), while an increase in the Munc13 concentration was shown in rats housed in an enriched environment. In conclusion, cocaine self-administration is associated with the specific up-regulation of the NMDA receptor subunit composition and is related with new presynaptic targets controlling neurotransmitter release. Moreover, changes observed in cocaine abstinence with extinction training and in an enriched environment in the levels of NMDA receptor subunit and in the active zone protein, respectively, may represent a potential regulatory step in cocaine-seeking behavior.

## 1. Introduction

Glutamate is the major excitatory neurotransmitter, mediating nearly 70% of synaptic transmission within the central nervous system. Glutamate is accumulated into synaptic vesicles and, upon depolarization of the presynaptic terminal is released into the synaptic cleft, then glutamate binds to various specific ionotropic and metabotropic glutamate receptors localized in almost all brain areas [1]. The ionotropic group of glutamate receptors is composed of *N*-methyl-d-aspartate (NMDA), amino-3-hydroxy-5-methyl-4-isoxazolepropionate (AMPA), and kainate receptors. NMDA receptors are heterotetramers composed of two obligatory GluN1 subunits and two accessory GluN2 (A-D) or rarely GluN3 subunits [2]. NMDA receptors require binding of both glutamate and the co-agonist glycine, as well as membrane depolarization for removal of a magnesium block and activation of these receptors results in the opening of channel pore for sodium, magnesium and calcium [3]. Stimulation of the glutamatergic receptors drives synaptic phenomena including several intracellular signaling pathways, leading to neuronal plasticity.

In fact, cocaine-induced plasticity in glutamatergic transmission is critically involved in the development of substance use disorder [4,5]. Several lines of evidence have demonstrated that cocaine exposure modulates glutamate levels, receptors and transporter expression [2,6]. Cocaine blocks the dopamine transporter in the plasma membrane of the accumbal dopamine nerve terminal networks and increases dopamine levels, which mediates the rewarding effects of cocaine, followed by activation of dopamine D_1_ and D_2_ receptors within the terminal areas of the mesolimbic neurons [7,8]. Dopamine can indirectly modulate the NMDA-mediated glutamate signaling via postsynaptic D_1_ receptors [9,10]. In addition to inhibiting dopamine uptake, cocaine also interacts with the serotonin transporter, which provides an initial and sustained cocaine self-administration in mice with deletion of the dopamine transporter [11].

Corticostriatal synaptic plasticity includes functional plasticity [long-term depression (LTD) and long-term potentiation (LTP)] and structural plasticity (changes in dendritic spine numbers, the thickness of postsynaptic density and changes in the active zone proteins) [12]. NMDA receptors participate in synaptogenesis and synapse plasticity in the nervous system under physiological and pathophysiological conditions; accordingly, changes in the composition of NMDA receptor subunits (GluN2B and GluN2A) result in different forms of LTP and LTD at excitatory synapses [13]. Many of the active zone proteins drive regulated secretion of synaptic vesicles in neurons, i.e., Ras-related protein 3A (Rab3A), Rab3 interacting molecules 1 (RIM1) and mammalian uncoordinated protein 13 (Munc13). Rab3A is a small GTP binding protein of the Ras gene superfamily localized to synaptosomes and secretory granules [14]. RIM1 is a putative effector for the synaptic vesicle protein Rab3A, playing a significant role in regulated secretion and neurotransmitter release [14]. Munc13 plays a role in vesicle maturation during neurotransmitter release by acting in synaptic vesicle priming prior to vesicle fusion, it is particularly important in most glutamatergic-mediated synapses [15]. It has been shown that RIM1 and Munc13 form a trimer with Rab3A [16]. This complex links synaptic vesicles to cytomatrix and places synaptic vesicles near the presynaptic plasma membrane, which in turn can modulate the neurotransmitters release and synaptic transmission intensity, as well as NMDA receptor membrane expression [16].

**Figure 1:**
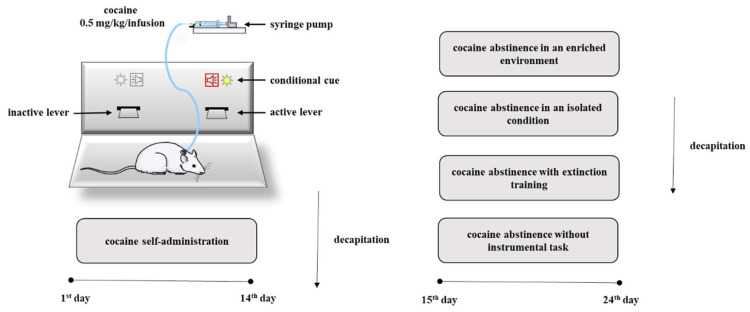
Diagram illustrating the experimental procedure.

Given the critical role in the release of neurotransmitter for the action of drugs of abuse, we decided to investigate changes in the levels of NMDA receptor subunit expression and in the levels of Rab3A, RIM1 and Munc13 proteins in the rat nucleus accumbens in association with compulsive cocaine self-administration, as well as in a different condition of cocaine forced abstinence. To better define the influence of abstinence on the mechanisms regulating the release of neurotransmitters, cocaine abstinence was performed under different conditions such as cocaine abstinence in an isolated condition, cocaine abstinence in an enriched environment, cocaine abstinence with extinction training or without instrumental task (Figure 1).

## 2. Results

### 2.1. Behavioral Effects

#### 2.1.1. Cocaine Self-Administration

**Figure 2:**
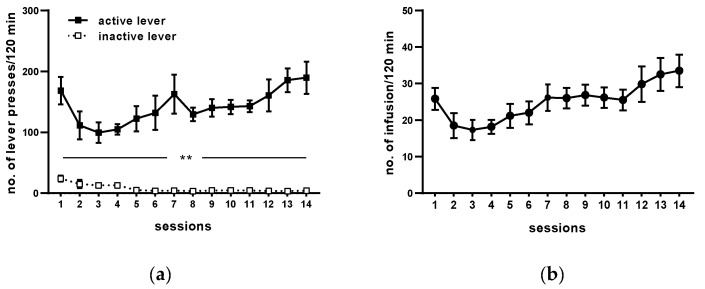
Maintenance of cocaine (0.5 mg/kg/infusion) self-administration (representative graph). All data are expressed as means ± SEM. N = 6 rats/group. ** *p* < 0.01 vs. inactive lever. (**a**), numbers of active and inactive lever presses; (**b**), numbers of cocaine infusions.

Following 14 sessions, rats acquired cocaine self-administration (i.e., they received >23 infusion/2 h under 0.5 mg/kg/infusion) and displayed <10% variation in the number of cocaine infusions (Figure 2). The mean number of cocaine infusions per day during the last five self-administration days varied from 23 to 29. The mean of total cocaine intake during 14 days for active cocaine group was from 148 ± 7 to 203 ± 9.81 mg/rat (Figure 2 and Table 1).

During 14 days of cocaine self-administration, rats pressed significantly more frequently on the active than on the inactive lever from the 1st till 14th day of self-administration (*p* < 0.01; Figure 2). During the last 5 days of training, daily cocaine intake was stable (F(4, 25) = 0.821; *p* = 0.524), as well as behavioral responses, readily discriminated between the inactive and active lever (lever: F(1, 10) = 153.525, *p* < 0.001; day: F(4, 40) = 0.380, *p* = 0.822; lever × day: F(4, 40) = 0.444 *p* = 0.776). The yoked saline and cocaine groups of rats had no difference in pressing the active vs. the inactive lever observed (yoked saline: lever F(1, 10) = 0.013, *p* = 0.912; day F(4, 40) = 0.818, *p* = 0.521; lever × day F(4, 40) = 0.52, *p* = 0.721; yoked cocaine: lever F(1, 10) = 1.046, *p* = 0.33; day F(4, 40) = 2.181, *p* = 0.089; lever × day F(4, 40) = 0.356, *p* = 0.838).

#### 2.1.2. Cocaine Abstinence in an Enriched Environment

During the maintenance of self-administration, daily cocaine intake was stable by the last 5 days of training (F(4, 35) = 0.259; *p* = 0.902) and rats presented stable behavioral responses on levers, readily discriminating between the active and inactive lever (lever (F1, 14 = 25.652; *p* < 0.000; day F(4, 56) = 1.023, *p* = 0.403; day × lever F(4, 56) = 0.906; *p* = 0.466). In the yoked cocaine (lever (F(1, 14) = 0.298, *p* = 0.593; day F(4, 56) = 1.944, *p* = 0.115; day × lever F(4, 56) = 0.729, *p* = 0.575) and yoked saline (lever (F(1, 14) = 1.963, *p* = 0.183; day F(4, 56) = 0.536, *p* = 0.710; day × lever F(4, 56) = 0.303, *p* = 0.875) groups, no difference in pressing the active versus the inactive lever was observed (Table 1).

#### 2.1.3. Cocaine Abstinence in an Isolated Condition

During the maintenance of self-administration, daily cocaine intake was stable by the last 5 days of training (F(4, 30) = 0.048; *p* = 0.995) and rats presented stable behavioral responses on levers, readily discriminating between the active and inactive lever (lever (F(1, 12) = 19.790, *p* < 0.000; day F(4, 48) = 1.323, *p* = 0.275; day × lever F(4, 48) = 0.270, *p* = 0.896). In the yoked cocaine (lever (F(1, 12) = 0.499, *p* = 0.493; day F(4, 48) = 1.996, *p* = 0.110; day × lever F(4, 48) = 0.542, *p* = 0.705) and yoked saline (lever (F(1, 12) = 1.998, *p* = 0.183; day F(4, 48) = 1.210, *p* = 0.319; day × lever F(4, 48) = 1.182, *p* = 0.330) groups, no difference in pressing the active versus the inactive lever was observed (Table 1).

#### 2.1.4. Cocaine Abstinence with Extinction Training

During the maintenance of self-administration daily cocaine intake was stable by the last 5 days of training (F(4, 35) = 0.539; *p* = 0.707) and rats presented stable behavioral responses on levers, readily discriminated between the active and inactive lever (lever (F(1, 14) = 82.265, *p* < 0.001; day F(4, 56) = 2.568, *p* = 0.047; day × lever F(4, 56) = 1.720, *p* = 0.158. In the yoked cocaine (lever (F(1, 14) = 0.107, *p* = 0.749; day F(4, 56) = 3.244, *p* < 0.01; day × lever F(4, 56) = 0.158, *p* = 0.958) and yoked saline (lever (F(1, 14) = 0.858, *p* = 0.369; day F(4, 56) = 1.747, *p* = 0.152; day × lever F(4, 56) = 0.505, *p* = 0.732) groups, no difference in pressing the active versus the inactive lever was observed (Table 1). All animals met the extinction criterion (i.e., responses on the active lever fell to <20% of the responses at the active lever reached during self-administration of cocaine) (i.e., the mean number of presses on the active and inactive levers in the last three days of the extinction training group was 25 ± 3.04 and 8.17 ± 2.34, respectively (17% of total presses vs. cocaine self-administration)).

#### 2.1.5. Cocaine Abstinence without the Instrumental Task

During the maintenance of self-administration daily cocaine intake was stable by the last 5 days of training (F(4, 35) = 0.259; *p* = 0.902), and rats presented stable behavioral responses on levers, readily discriminated between the active and inactive lever (lever (F(1, 14) = 21.215, *p* < 0.001; day F(4, 56) = 0.640, *p* = 0.283; day × lever F(4, 56) = 0.640, *p* = 0.635). In the rats passively self-administering cocaine (lever (F(1, 14) = 1.570, *p* = 0.230; day F(4, 56) = 0.282, *p* = 0.888; day × lever F(4, 56) = 0.037, *p* = 0.997) and saline (lever (F(1, 14) = 1.128, *p* = 0.306; day F(4, 56) = 1.237, *p* = 0.306; day × lever F(4, 56) = 0.886, *p* = 0.478), there was no difference between pressing the active lever versus the inactive lever (Table 1).

When they compare cocaine abstinence in different conditions, there was no effects between groups self-administering cocaine (day × group × lever F(12, 216) = 0.805; *p* = 0.645), the yoked cocaine groups (day × group × lever F(12, 216) = 0.708; *p* = 0.742), and the yoked saline groups (day × group × lever F(12, 216) = 0.972; *p* = 0.476) (Table 1).

### 2.2. Expression of NMDA Receptor Subunits

#### 2.2.1. Cocaine Self-Administration

**Figure 3:**
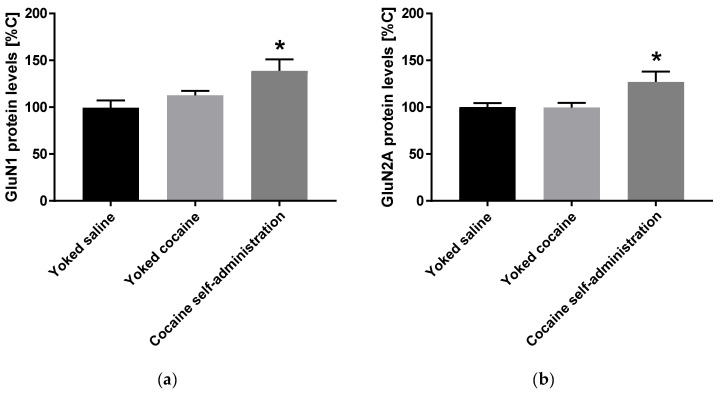

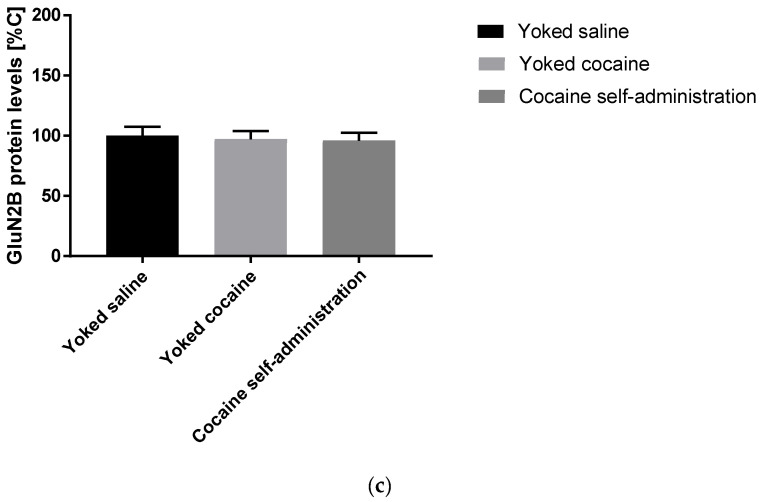
Changes in the expression of NMDA receptor subunits (GluN1, GluN2A and GluN2B) in the nucleus accumbens in rats following cocaine self-administration. All data are expressed as % of control (mean ± SEM). N = 6 rats/group. * *p* < 0.05 vs. yoked saline. (**a**), GluN1 protein levels [% of control]; (**b**), GluN2A protein levels [% of control].; (**c**), GluN2B protein levels [% of control].

An increase in the expression of accumbal GluN1 (F(2, 15) = 5.258; *p* = 0.019) and GluN2A (F(2, 15) = 4.621; *p* = 0.027) subunits was observed in rats self-administered cocaine, while the expression of GluN2B subunit did not change after cocaine self-administration (Figure 3).

#### 2.2.2. Cocaine Forced Abstinence

Cocaine abstinence in an isolated condition and in an enriched environment did not change the levels of NMDA receptor subunit in the nucleus accumbens in rats that previously self-administered cocaine (Table 2; all membranes are presented in the Supplementary Materials).

Cocaine abstinence with extinction training in rats self-administering cocaine only induced a rise in the GluN1 subunit expression levels in the nucleus accumbens (F(2, 21) = 3.555; *p* = 0.047) (Table 2; all membranes are presented in the Supplementary Materials).

The 10-daily cocaine abstinence without instrumental task in rats actively as well as passively administering cocaine did not change the levels of NMDA receptor subunits (Table 2; all membranes are presented in the Supplementary Materials).

### 2.3. Proteins Involved in Synaptic Vesicle Exocytosis

#### 2.3.1. Cocaine Self-Administration

**Figure 4:**
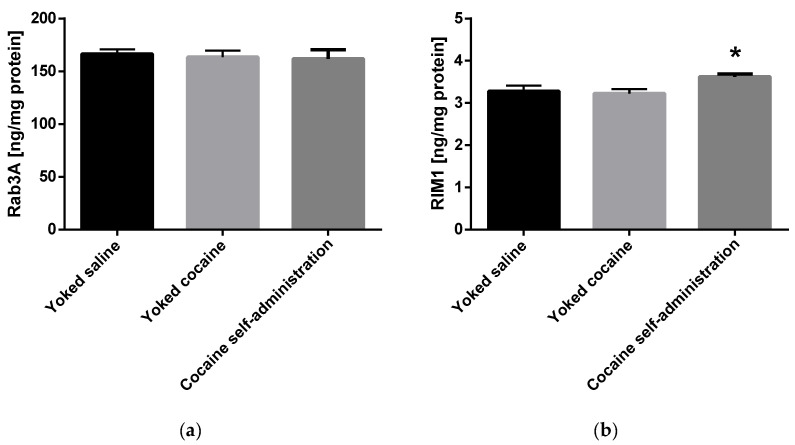

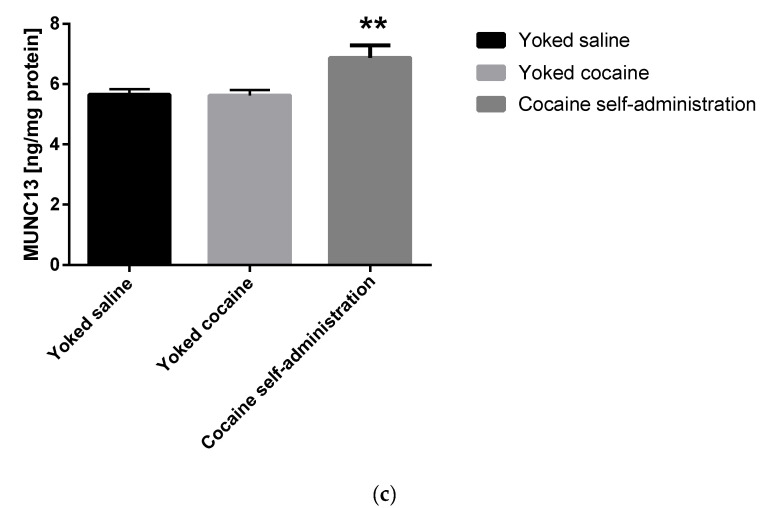
Changes in the concentration of Rab3A, RIM1 and Munc13 in the nucleus accumbens in rats following cocaine self-administration. Ras-related protein A (Rab3A), Rab3 interacting molecules 1 (RIM1) and mammalian uncoordinated protein 13 (Munc13). All data are expressed as mean ± SEM. N = 6 rats/group. * *p* < 0.05 vs. yoked saline. (**a**), Rab3A [ng/mg protein]; (**b**), RIM1 [ng/mg protein]; (**c**), Munc13 [ng/mg protein].

An increase in the Munc13 (F(2, 15) = 6.478; *p* = 0.009) and RIM1 (F(2, 15) = 4.294; *p* = 0.034) level was shown in rats self-administering cocaine in the nucleus accumbens (Figure 4).

#### 2.3.2. Cocaine Forced Abstinence

Enriched environment evoked a rise only in the Munc13 concentration (F(2, 21) = 3.807; *p* = 0.039) in the nucleus accumbens (Table 3).

In other conditions of cocaine abstinence, the levels of proteins involved in synaptic vesicle exocytosis did not change in rats previously administered cocaine (Table 3).

It should also be noted that different conditions of cocaine abstinence did not change the basal levels of accumbal proteins involved in synaptic vesicle exocytosis in yoked saline group (*Rab3A*: F(3, 27) = 2.074; *p* = 0.127; *RIM1*: F(3, 27) = 2,227; *p* = 0.108; *Munc13*: F(3, 27) = 1.256; *p* = 0.309).

## 3. Discussion

In this study, we found that cocaine-self-administration caused the up-regulation of the accumbal levels of GluN1 and GluN2A subunits of the NMDA receptor accompanied with higher levels of Munc13 and RIM1 proteins. At the same time, we also found that different conditions of cocaine abstinence abolished changes related to NMDA receptor subunits, while an increase in the Munc13 concentration was shown in rats housed in an enriched environment. Moreover, an increase in the GluN1 subunit expression was maintained in the animals previously self-administering cocaine after 10-day cocaine abstinence with extinction training.

Cocaine use disorder is mediated by experience-dependent synaptic plasticity in the nucleus accumbens [9]. The nucleus accumbens is the structure closely tied to motivational mechanisms [17] and receives excitatory glutamatergic inputs from the hippocampus, amygdala, and prefrontal cortex [18], as well as dopaminergic innervation from the ventral tegmental area, which modulates glutamatergic transmission and mediates the cocaine rewarding effects [5]. It should be noted that these glutamatergic inputs seem to be crucial for the learning and maintenance of drug-seeking behavior [19]. Changes in NMDA receptor subunit composition may underline the transition from the occasional use of cocaine to substance use disorder. The development of drug craving by enhancing the incentive motivational value of cocaine is accompanied by enduring different neuronal changes within glutamate signaling. In fact, an increase in the expression of GluN1 subunit, which is the obligatory subunit in the NMDA receptors, and GluN2A subunit was observed in our study in rats self-administering cocaine. The latter changes may lead to the increased excitability due to increased calcium flux through NMDA receptors in this brain structure which, in turn, may induce long-term biochemical and behavioral effects of cocaine. Additionally, an increase of the accumbal NMDA receptor subunits levels may also constitute a compensatory up-regulation in response to reduced basal synaptic concentrations of glutamate [20]. In fact, during repeated cocaine self-administration protocol, cocaine evokes small increases of glutamate extracellular levels at a few isolated time points in the nucleus accumbens of animals previously trained to self-administer cocaine [21] while decreasing basal extracellular levels of accumbal glutamate [20,22]. Based on preclinical studies, it was shown that acute and repeated experimentally delivered cocaine administration reduced the GluN1 mRNA level in the nucleus accumbens [23,24]. Conversely, in rhesus monkeys self-administering cocaine, the increased expression of this subunit was shown [25]. It was also shown that GluN1 mRNA levels were initially increased following cocaine self-administration, an effect that progressively decreased following 1, 5, or 10 days of extinction in the nucleus accumbens [26]. Such increased glutamate signaling has also been shown in post mortem samples from cocaine addicts after overdose [25]. Therefore, increased glutamatergic neurotransmission manifested by higher levels of GluN1 and GluN2A subunits seen in our study probably reflect a motivational aspect of self-administered cocaine.

In our study, changes in the expression of NMDA receptor subunits were abolished after different condition of cocaine abstinence. Only in rats extinguished from cocaine self-administration, was an increase in the GluN1 subunit expression observed. The increase in GluN1 levels in the cocaine abstinence with extinction training in rats previously self-administered cocaine might be also related with the motivation-related learning and memory. In fact, extinction training can be considered a form of learning that involves the formation of a new memory that suppresses behavioral responses to a learned stimulus. Therefore, the increase of GluN1 only in this group of cocaine abstinence might reflect this form of new learning. The latter change in the nucleus accumbens—seen also immediately after cocaine self-administration—was more persistent and evident even after ten days of cocaine abstinence. It was shown that repeated cocaine injections increased the accumbal level of GluN1 subunits 3 weeks after cocaine abstinence [27], while in rats subjected to extinction procedures, a decrease in GluN1 expression in the nucleus accumbens shell [28] and core was observed [29]. As discussed previously for rats during cocaine self-administration, it has been shown that increased levels of GluN1 subunit may reflect a compensatory mechanism for decreased basal levels of glutamate [20]. In our previous study, using the same behavioral protocol, we showed that 10-day extinction training reduced basal glutamate levels in the nucleus accumbens as compared with baseline of saline-yoked controls [20]. In contrast to the present study, a rise in both GluN2A and GluN2B subunits levels was reported in rats housed in their home cage [30]. However, the differences in experimental conditions, like dose of self-administered cocaine (0.5 versus 0.25 mg/kg/infusion), fixed-ratio schedule of reinforcement (FR5 versus FR1), session length (120 versus 90 min), duration of extinction (10 versus 14 days), the way of euthanizing (decapitation versus anesthetization with isoflurane gas), and sensitivity of animal strain (Wistar versus Long-Evans rats) may help explain, at least partially, such discrepancies.

Cocaine enhances dopaminergic synaptic transmission by blockade of the dopamine transporter (DAT), which in turn leads to elevation of extracellular dopamine levels in the nucleus accumbens [31]. In our previous study, it was also shown that cocaine self-administration elevated extracellular accumbal dopamine levels during last cocaine session, whereas it did not affect basal accumbal dopamine levels [20]. Therefore, increased levels of proteins (Munc13 and RIM1) were probably related to exocytosis of other neurotransmitters rather than glutamate. In fact, the results suggest that cocaine self-administration enhanced synaptic transmission efficiency by increasing Munc13 and RIM1 protein levels in the nucleus accumbens. Since RIM1, Munc13 and Rab3A exist in all synaptic terminals, further research is needed to explain these phenomena. Increased RIM1 levels were consistent with the known annotation of these proteins to calcium availability, transport processes and calcium channel activity [32,33]. RIM1 is involved in the facilitation of neurotransmitter release through the regulation of voltage-gated calcium channels, L type, alpha 1D subunit (Cav1.3) by G-proteins [33]. Enhanced Cav1.3 channel activity was reported in the mouse nucleus accumbens after cocaine conditioned place preference test, and it was emphasized that this up-regulation seems to be one potential mechanism underlying drug abuse and mood disorders [32]. On the other hand, the level of D2 receptors and Cav1.3 channels in the dorsomedial striatum was reduced in the well-established habitual-seeking behavior in animals [34]. In turn, D2 receptors activate Cav1.3 to suppress its downstream activation (CREB, dopamine and cAMP-regulated phosphoprotein, Protein Kinase B (Akt) and Glycogen synthase-3 (GSK-3)) [34]. Thus, increased levels of RIM1 protein concentration may reflect compensatory mechanism for depressed activity of Cav1.3 channels in rats self-administered cocaine. Recently, over-expression of RIM1 genes in the amphetamine overactive mice was found in the striatum [35].

Little is known about the effect of cocaine on the Munc13 levels in the rat brain. Munc13 is the protein involved in priming and docking of synaptic vesicles [36]. As a priming factor, Munc13 contains at least three regulatory domains that are activated directly or indirectly by calcium [37]. Increased accumbal levels of Munc13 were seen in rats following cocaine self-administration, as well as following cocaine abstinence in an enriched environment. Considering the fact that the basal level of Munc13 was not changed under different conditions of cocaine abstinence in yoked saline (control) rats, as well as that Munc13 levels were not altered in rats previously administered cocaine in other cocaine abstinence procedures than rats housed in an enriched environment, one can suppose that increased level of Munc13 is probably as a result of the combination of an enriched environment and cocaine and enriched environment enhances the cocaine-induced motivation-driven learning. After abstinence in an enriched environment, a reduction in drug seeking behavior to the conditioned stimulus [38,39] and cocaine priming [39] was reported. This type of drug forced abstinence—in contrast to extinction training under the contexts/environments where the original memories were formed—makes extinction memories generalize to other contexts. Cocaine seeking behavior is associated with main cognitive processes such as attention, memory and decision making. It was shown that the enriched environment itself may serve as a salient natural reward for reduction of cocaine seeking and cocaine taking. In fact, rats housed in an enriched environment continued to avoid intake of the cocaine-associated saccharin cue [40]. Moreover, it was shown that extinction training and enriched environment attenuated cue-induced cocaine-seeking behavior [39,41], while the combined therapy using the extinction training with an enriched environment completely prevented it [41]. Environmental enrichment differentially regulates the response to psychostimulants in the nucleus accumbens shell and core by modulation of DAT function [42]. In fact, in rats housed in enriched conditions relative to impoverished conditions, dopamine clearance was reduced in the nucleus accumbens shell, while clearance increased in the nucleus accumbens core [42]. Therefore, increased accumbal levels of Munc13 seem to be related with dopamine release in rats following cocaine self-administration, as well as following cocaine abstinence in an enriched environment. In turn, dopamine can modulate the glutamatergic neurotransmission and NMDA receptor-dependent synaptic plasticity via postsynaptic D1 receptors. Activation of D1 receptors induces a fast lateral redistribution of the NMDA receptors, increases NMDA receptor synaptic content and favors long-term synaptic potentiation [9,10]. On the other hand, it was shown that Munc13 is an essential protein for synaptic vesicle maturation in glutamatergic hippocampal neurons in mice lacking Munc13 [43], and the higher level of this protein may be involved in glutamate release during reinstatement of cocaine-seeking.

Additionally, neither cocaine self-administration nor drug abstinence changed the level of Rab3A in the nucleus accumbens. It should be noted that Rab3A does not appear to be obligatory for synaptic vesicle trafficking to the active zone per sé, but rather modulates the release of neurotransmitters by ameliorating the release probability of a subset of primed vesicles [44].

In conclusion, our present findings indicate that cocaine self-administration is associated with up-regulation of the NMDA receptor subunit composition and enhanced presynaptic mechanisms of neurotransmitter release as shown by changes in the levels of proteins within active zone. Moreover, changes observed in cocaine abstinence with extinction training and in an enriched environment in the levels of NMDA receptor subunit and in the active zone protein, respectively, may represent a potential regulatory step in cocaine-seeking behavior. Our research provides further evidence that both changes observed during cocaine abstinence with extinction training or housed in an enriched environment may represent a potential method for treating cocaine relapse; however, combining therapy approaches (i.e., cognitive, viral-based or genetic therapy) may improve outcomes.

## 4. Materials and Methods

### 4.1. Animals

Male Wistar rats (225–250 g; Charles River, Sulzfeld, Germany) were housed in collective cages in a room maintained at 22 ± 2 °C and 55 ± 10% humidity under a 12-h light–dark cycle (between 6 a.m. and 6 p.m.) with free access to water and standard animal food (VRF1 pellets, UK), except for the initial training of lever presses and the first three days of self-administration procedure, see below. After intravenous catheter implantation and during the recovery period, or self-administration procedures, animals were kept individually in standard plastic rodent cages. All self-administration sessions were completed at the same time each day, between 7:30 a.m. and 3 p.m. daily, depending on the experiment. All the experiment procedures were carried out in accordance with EU directive 2010/63/EU and with approval of the Local Ethics Commission at the Maj Institute of Pharmacology Polish Academy of Sciences in Krakow, Poland (1235/2015).

### 4.2. Drugs

Cocaine hydrochloride (Toronto Research Chemicals, North York, ON, Canada) was dissolved in sterile 0.9% NaCl and given intravenously in a volume of 0.1 mL per infusion.

### 4.3. Intravenous Catheter Implantation

Rats were anesthetized with a mixture of ketamine hydrochloride (75 mg/kg, i.m.; Biowet, Puławy, Poland) and xylazine (5 mg/kg, i.m.; Biowet, Puławy, Poland) and implanted with a silastic catheter in the external right jugular vein, as described previously [45]. During 3 days after surgery, meloxicam (Metacam, Boehringer IIngelheim, Ingelheim, Germany; 5 mg/kg, s.c.) was used to reduce post-operative pain. All animals had a recovery period of at least 7 days, during which catheters were flushed daily with 0.2 mL of saline solution containing cephazolin (100 mg/mL; Biochemie GmbH, Kundl, Austria) and heparin (100 U/mL; Biochemie GmbH, Kundl, Austria) to prevent catheter non-patency as a result of blood clotting.

### 4.4. Initial Training to Lever Presses

Before self-administration training, rats were trained to press the lever for food pellets (VRF1 pellets, UK) under a fixed ratio (FR) from one to five reinforcement schedule. Food training and self-administration sessions were performed in a sound attenuated, standard operant conditioning chambers (Med-Associates, St. Albans, VT, USA) from 2 to 3 days for 2 h daily. Starting 24 h prior to the food training session, rats received rations of ~20 g of chow daily. Each chamber was equipped with a reward feeder; presses on the active lever resulted in the delivery of 0.1 mL of sweetened milk, which continued until rats reached a criterion of 100 active lever presses. The inactive lever was not programmed.

### 4.5. Cocaine Self-Administration

Following the food training period, rats began the self-administration procedures using the same standard operant chambers. The criterion for cocaine self-administration was a minimum of ten infusions per day (0.5 mg/kg/infusion, 2 h/day) for 14 days. Active lever presses during cocaine self-administration resulted in delivery of a dose of cocaine, as well as the simultaneous stimuli of light illumination (24-V) above the active lever and a tone presentation (2000 Hz; 15 dB) for a programmed duration of 5 s. A 20-s timeout followed the delivery of each infusion during which time active lever presses were recorded but had no consequences. Presses on the inactive lever were recorded, but not reinforced. Acquisition of the conditioned operant response lasted a minimum of 5 days until subjects met a stable average for three consecutive days and a standard deviation within those days of <10% of the average [46]. After the 14th (2-h) self-administration session, a subset of the above cohort (N = 6 rats/group) was decapitated. The remaining cohort trained to self-administer cocaine (0.5 mg/kg/infusion) for 14 days was exposed to the four abstinence procedures (see Section 4.7.). The experimental protocol steps are presented in Figure 1.

### 4.6. ‘Yoked’ Procedures

For biochemical experiments, the yoked procedure was employed. In this procedure, rats were tested simultaneously in groups with rats serving as yoked controls that received an injection of saline or cocaine, which was not contingent on the response, and each time a response-contingent injection of 0.5 mg/kg cocaine was self-administered by the paired rat. Unlike self-administering rats, lever pressing by the yoked rats was recorded but had no programmed consequence.

### 4.7. Cocaine Abstinence Procedures

After self-administration (once the rats met the maintenance criterion, see above) the rats trained to self-administer cocaine were separated to undergo 10 days of cocaine abstinence under four housing conditions: (i) an enriched environment; (ii) an isolated condition; (iii) cocaine abstinence with extinction training; and (iv) cocaine abstinence without the instrumental task.

#### 4.7.1. Cocaine Abstinence in an Enriched Environment

Enriched housing animals (actively self-administering, yoked cocaine, yoked saline; N = 8 rats/group) lived in standard large rat cages that housed four or five rats and contained bedding, two water bottles, short or long PVC pipes, pieces of cotton material mounted to the top of the cage, and small plastic and/or wood toys; these rats were handled several times per day. Toys, cotton material, and PVC pipes were changed three times per week to maintain novelty. All animals were taken straight out of the enriched environment and decapitated in the 10th cocaine abstinence day.

#### 4.7.2. Cocaine Abstinence in an Isolated Condition

During abstinence, rats were in the social isolation. To reduce social interactions, the animals (actively self-administering, yoked cocaine, yoked saline; N = 7 rats/group) lived individually in the plastic cage with white walls (isolation cage) in a room to which only the experimenter had access and animals were handled once per week. All animals were taken straight out of their home cages and decapitated in the 10th cocaine abstinence day.

#### 4.7.3. Cocaine Abstinence with Extinction Training

The three groups of rats (actively self-administering, yoked cocaine, yoked saline; N = 8 rats/group) following the last cocaine self-administration session underwent an extinction period. During extinction, all animals at 2-h daily training sessions had no delivery of cocaine or the presentation of the conditioned stimulus. Only animals that met the extinction criterion (i.e., responses on the active lever fell to <20% of the responses at the active lever reached during self-administration of cocaine) were sacrificed immediately following the last (10th) session of extinction training.

#### 4.7.4. Cocaine Abstinence without the Instrumental Task

During abstinence without the instrumental task, the animals (actively self-administering, yoked cocaine, yoked saline; N = 8 rats/group) following the last cocaine self-administration session underwent 10 days withdrawn in self-administered operant chambers, where the rats had no presentation of the conditioned stimulus and levers (only home light) during 2-h daily sessions. All animals were sacrificed immediately following the last (10th) session.

### 4.8. Dissection

After decapitation, the brain was quickly removed and chilled in ice-cold saline. The nucleus accumbens (shell and core; Bregma 2.2–1.0 mm) from each animal were dissected out [47]. Samples were immediately frozen on dry ice and stored at −80 °C for biochemical analyses.

### 4.9. Biochemical Analyses

#### 4.9.1. NMDA Receptor Subunits Analyses—Western Blot

Frozen brain structures were homogenized in cold 0.32 M sucrose buffer pH 7.4 containing 1 mM HEPES, 1 mM MgCl_2_, 1 mM NaHCO_3_ and 0.1 mM PMSF, in presence of cocktails of protease and phosphatase inhibitors (Sigma-Aldrich, St. Louis, MO, USA) using a in a teflon-glass potter. For protein determination, a bicinchoninic acid assay (BCA) protein assay kit (Serva, Heidelberg, Germany) was used. Homogenates (10 μg of proteins) were then denatured in SDS-PAGE sample buffer (62.5 mM Tris-HCl, pH 6.8, 10% glycerol, 2% SDS, and 0.001% bromophenol blue) containing 5% β-mercaptoethanol for 2 min at 85 °C, 2 min in ice, 5 min at 85 °C, and finally 2 min in ice. Protein samples were resolved by a gradient 4–16% SDS polyacrylamide gels and transferred to a polyvinylidene difluoride (PVDF) membrane. Membranes were blocked in 3% non-fat dry milk, and separate sets of membranes were probed with mouse anti-GluN1 monoclonal antibody (1:1000; 32-0500, Thermo Fisher Scientific, Waltham, MA, USA), rabbit anti-GluN2A polyclonal antibody (1:1000; A-6473; Molecular Probes, Leiden, The Netherlands), and rabbit anti-GluN2B polyclonal antibody (1:1000; ab65783; Abcam, Cambridge, UK). The expressions of NMDA receptor subunits were evaluated relative to that of β-actin control protein using mouse monoclonal antibody (1:1000, A5441; Sigma-Aldrich, St. Louis, MO, USA). Blots were washed and incubated with donkey goat anti-rabbit secondary antibody (1:6000; 926-68071; Li-cor, Lincoln, NE, USA) or goat anti-mouse (1:6000; 926-32210; Li-cor, Lincoln, NE, USA) and visualized with a fluorescence detection Odyssey Clx (Li-cor, Lincoln, NE, USA). Analysis was performed using Image Studio v.2.1. All data were expressed as % of control.

#### 4.9.2. RIM1, Munc13 and Rab3A Analyses—ELISA

Quantitative measurement of RIM1, Munc13 and Rab3A in tissue homogenates was performed using Rat Regulating synaptic membrane exocytosis protein 1 (RIM1) ELISA Kit (CK-bio-20387; Shanghai Coon Koon Biotech Co., Shanghai, China), Rat Protein unc-13 (Munc13) ELISA Kit (CK-bio-20385; Shanghai Coon Koon Biotech Co., Shanghai, China) and Rat Ras-related protein Rab3A (Rab3A) ELISA Kit (CK-bio-20386; Shanghai Coon Koon Biotech Co., Shanghai, China) following the manufacturer’s protocol. First, homogenates obtained for Western Blot analysis were centrifuged for 20 min at 1000× *g*. Supernatants were immediately removed, and protein concentration was determined in each sample with BCA protein assay kit. 100 µg of protein from each sample was used in the ELISA assays. All data were expressed as concentration ng/mg of protein.

### 4.10. Statistical Analyses

All data are expressed as the mean ± SEM. In behavioral experiments, the number of responses on the active and inactive lever and number of infusions were analyzed using one or two-way analysis of variance (ANOVAs) for repeated measurements, the latter analysis followed by post-hoc Newman-Keuls. In neurochemical studies, statistical analyses were performed with one-way ANOVA, followed by Dunnett’s test to analyze differences between group means. *p* < 0.05 was considered statistically significant.

## Figures and Tables

**Table 1:** Cocaine (0.5 mg/kg/infusion) self-administration. Presses on the levers and cocaine infusion.

Number of	Active Lever	Inactive Lever	Infusion
**Future cocaine abstinence in an enriched environment**			
Cocaine self-administration	197 ± 16.50	4 ± 0.57	28 ± 1.02
Yoked cocaine	10 ± 1.56	8 ± 0.79	
Yoked saline	12 ± 1.11	8 ± 0.32	
**Future cocaine abstinence in an isolated condition**			
Cocaine self-administration	203 ± 9.81	9 ± 5.13	29 ± 0.42
Yoked cocaine	38 ± 13.80	17 ± 5.34	
Yoked saline	18 ± 2.93	8 ± 0.72	
**Future cocaine abstinence with extinction training**			
Cocaine self-administration	145 ± 4.00	8 ± 2.39	26 ± 1.00
Yoked cocaine	2 ± 0.59	3 ± 0.42	
Yoked saline	5 ± 0.66	4 ± 0.65	
**Future cocaine abstinence without instrumental task**			
Cocaine self-administration	163 ± 4.68	11 ± 2.07	23 ± 1.31
Yoked cocaine	14 ± 5.46	7 ± 2.37	
Yoked saline	5 ±0.48	4 ± 0.44	

Data are presented as means ± SEM by the final 5 day of training.

**Table 2:** Changes in the expression of NMDA receptor subunits (GluN1, GluN2A and GluN2B) in the nucleus accumbens in rats following different conditions of cocaine forced abstinence.

NMDA Receptor Subunits	Yoked Saline	Yoked Cocaine	Cocaine Self-Administration	Statistical Analyses
**Cocaine abstinence in an enriched environment**				
**GluN1**	100 ± 10.9	92.2 ± 9.5	110.8 ± 11.0	F(2, 21) = 0.779; *p* = 0.472
**GluN2A**	100 ± 5.8	100.9 ± 7.3	92.8 ± 6.1	F(2, 21) = 0.476; *p* = 0.628
**GluN2B**	100 ± 8.0	91.8 ± 11.3	102.0 ± 15.9	F(2, 21) = 0.197; *p* = 0.822
**Cocaine abstinence in an isolated condition**				
**GluN1**	100 ± 14.1	92.0 ± 4.8	92.2 ± 14.9	F(2, 18) = 0.141; *p* = 0.869
**GluN2A**	100 ± 4.7	91.2 ± 10.6	97.6 ± 11.4	F(2, 18) = 0.235; *p* = 0.793
**GluN2B**	100 ± 3.5	94.4 ± 7.5	99.7 ± 6.5	F(2, 18) = 0.271; *p* = 0.766
**Cocaine abstinence with extinction training**				
**GluN1**	100 ± 3.2	129.3 ± 13.2	144.2 ± 15.6 *	F(2, 21) = 3.555; *p* = 0.047
**GluN2A**	100 ± 4.7	114.9 ± 10.9	129.1 ± 11.9	F(2, 21) = 2.234; *p* = 0.132
**GluN2B**	100 ± 5.2	108.5 ± 8.5	106.0 ± 10.6	F(2, 21) = 0.273; *p* = 0.764
**Cocaine abstinence without instrumental task**				
**GluN1**	100 ± 12.6	104.5 ± 21.8	101.9 ± 17.8	F(2, 21) = 0.016; *p* = 0.984
**GluN2A**	100 ± 6.7	113.2 ± 13	103.6 ± 10.7	F(2, 21) = 0.424; *p* = 0.660
**GluN2B**	100 ± 11.3	102.6 ± 14.2	106.1 ± 11.8	F(2, 21) = 0.061; *p* = 0.942

All data are expressed as % of control (mean ± SEM). N= 7–8 rats/group. * *p* < 0.05 vs. yoked saline.

**Table 3:** Changes in the concentration of Rab3A. RIM1 and Munc13 in the nucleus accumbens in rats following different conditions of cocaine forced abstinence: (a) cocaine abstinence in an enriched environment; (b) cocaine abstinence in an isolated condition; (c) cocaine abstinence with extinction training; or (d) cocaine abstinence without instrumental task.

Protein	Yoked Saline [ng/mg Protein]	Yoked Cocaine [ng/mg Protein]	Cocaine Self-Administration [ng/mg Protein]	Statistical Analyses
**Cocaine abstinence in an enriched environment**				
**Rab3a**	147.8 ± 5.4	135.4 ± 2.7	136.5 ± 4.7	F(2, 21) = 2.401; *p* = 0.115
**RIM1**	3.7 ± 0.2	3.4 ± 0.1	3.6 ± 0.2	F(2, 21) = 0.926; *p* = 0.412
**Munc13**	5.4 ± 0.1	5.3 ± 0.1	5.9 ± 0.3 *	F(2, 21) = 3.807; *p* = 0.039
**Cocaine abstinence in an isolated condition**				
**Rab3a**	143 ± 4.4	131.5 ± 3.2	137.9 ± 2.1	F(2, 18) = 2.869; *p* = 0.083
**RIM1**	4.2 ± 0.2	4.5 ± 0.2	4.3 ± 0.2	F(2, 18) = 1.049; *p* = 0.371
**Munc13**	6.1 ± 0.3	6.6 ± 0.2	6.6 ± 0.3	F(2, 18) = 0.446; *p* = 0.647
**Cocaine abstinence with extinction training**				
**Rab3a**	132 ± 5.1	137.1 ± 3.4	128.9 ± 3	F(2, 21) = 1.109; *p* = 0.349
**RIM1**	4.2 ± 0.2	4.6 ± 0.1	4.2 ± 0.1	F(2, 21) = 1.437; *p* = 0.26
**Munc13**	5.8 ± 0.2	6.3 ± 0.1	5.6 ± 0.2	F(2, 21) = 3.448; *p* = 0.051
**Cocaine abstinence without instrumental task**				
**Rab3a**	138.0 ± 3.8	130.9 ± 5.3	141.7 ± 4.4	F(2, 21) = 1.449; *p* = 0.257
**RIM1**	3.9 ± 0.1	3.8 ± 0.1	4.2 ± 0.2	F(2, 21) = 2.307; *p* = 0.124
**Munc13**	5.9 ± 0.4	5.6 ± 0.1	5.7 ± 0.2	F(2, 21) = 0.461; *p* = 0.636

Ras-related protein A (Rab3A). Rab3 interacting molecules 1 (RIM1) and mammalian uncoordinated protein 13 (Munc13). All data are expressed as mean ± SEM. N = 7–8 rats/group. * *p* < 0.05 vs. yoked saline.

## Supplementary Materials

The supplementary materials are available online.

## Abbreviations

Cav1.3	voltage-gated calcium channels, L type, alpha 1D subunit
FR	fixed ratio
LTD	long-term depression
LTP	long-term potentiation
Munc13	mammalian uncoordinated protein 13
NMDA	*N*-methyl-d-aspartate
Rab3A	Ras-related protein 3A
RIM1	Rab3 interacting molecules 1
