# Supplementary material for: Cocaine Self-Administration and Abstinence Modulate NMDA Receptor Subunits and Active Zone Proteins in the Rat Nucleus Accumbens
Source: Molecules. 2020 Jul 31;25(15):3480. doi: 10.3390/molecules25153480 (PMC7436251; doi:10.3390/molecules25153480)

COCAINE SELF-ADMINISTRATION AND ABSTINENCE MODULATE NMDA RECEPTOR SUBUNITS AND ACTIVE ZONE PROTEINS IN THE RAT NUCLEUS ACCUMBENS

Irena Smaga[a*], Karolina Wydra[a], Małgorzata Frankowska[a], Fabio Fumagalli[b], Marek Sanak[c], Małgorzata Filip[a]

[a]Maj Institute of Pharmacology Polish Academy of Sciences, Department of Drug Addiction Pharmacology, Smętna 12, PL 31-343 Kraków, Poland

[b]Department of Pharmacological and Biomolecular Sciences, Università degli Studi di Milano, Via Balzaretti 9, 20133 Milano, Italy

[c]Department of Internal Medicine, Jagiellonian University Medical College, Skawińska 8, PL 31-066 Kraków, Poland

* Corresponding author.

E-mail address: smaga@if-pan.krakow.pl

Tel.: +48 12 6623268, Fax: +48 12 6374500

Supporting Information Table 3. Corresponding membranes from Western blot analyses of NMDA receptor subunits and loading controls. YS- yoked saline, YC- yoked cocaine, SA-cocaine self-administration. Control protein (β-actin) for GluN1 and GluN2B was the same.

<table>
<tr><td>NMDA receptor subunits</td><td>Membranes</td></tr>
<tr><td colspan="2">Cocaine abstinence in an enriched environment</td></tr>
<tr><td>GluN1</td><td>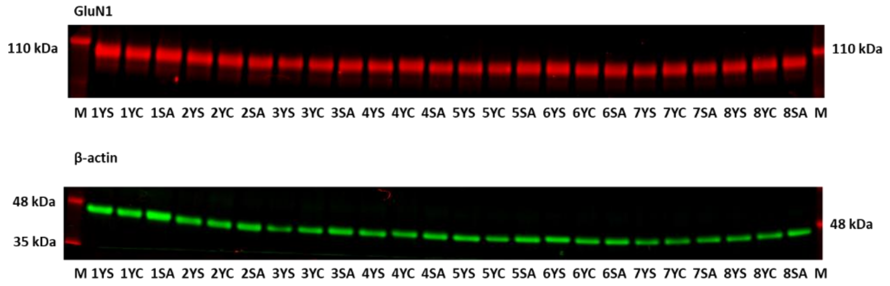
</td></tr>
<tr><td>GluN2A</td><td>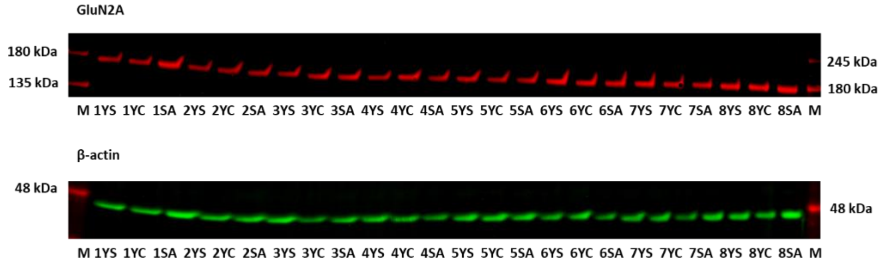
</td></tr>
<tr><td>GluN2B</td><td>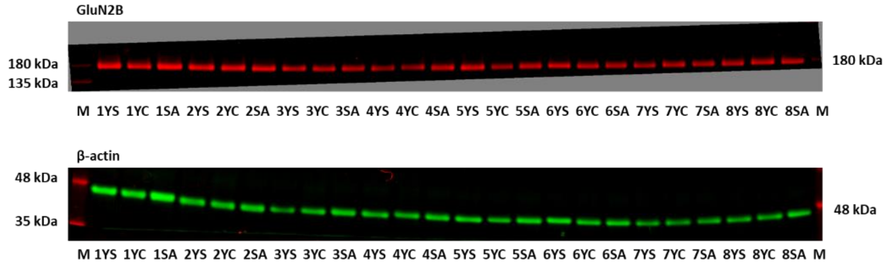
</td></tr>
<tr><td colspan="2">Cocaine abstinence in an isolated condition</td></tr>
<tr><td>GluN1</td><td>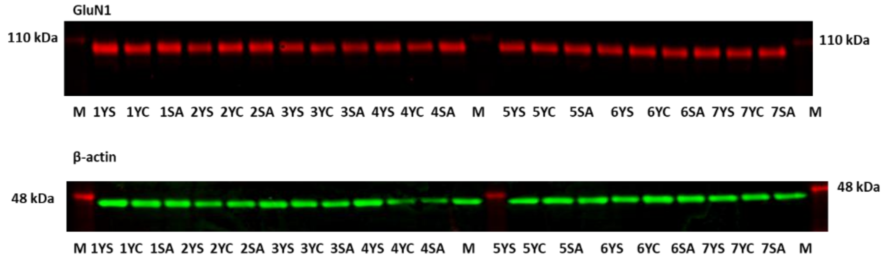
</td></tr>
</table>

<table>
<tr><td>GluN2A</td><td>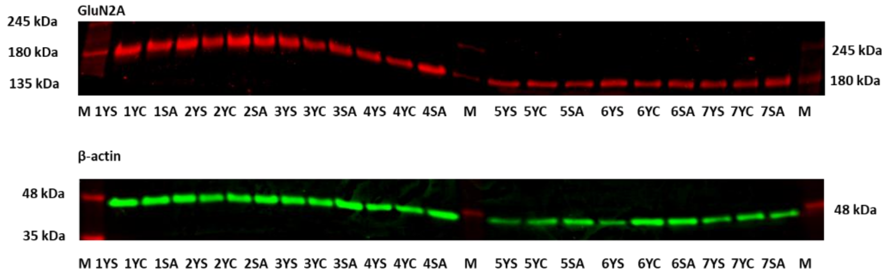
</td></tr>
<tr><td>GluN2B</td><td>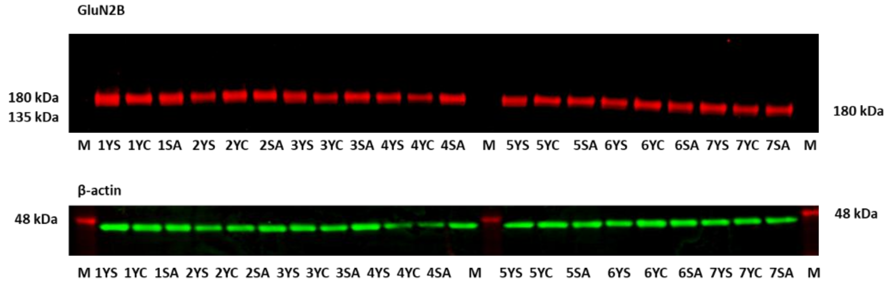
</td></tr>
<tr><td colspan="2">Cocaine abstinence with extinction training</td></tr>
<tr><td>GluN1</td><td>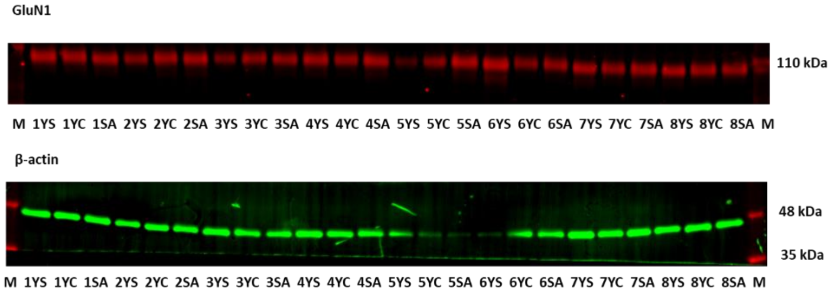
</td></tr>
<tr><td>GluN2A</td><td>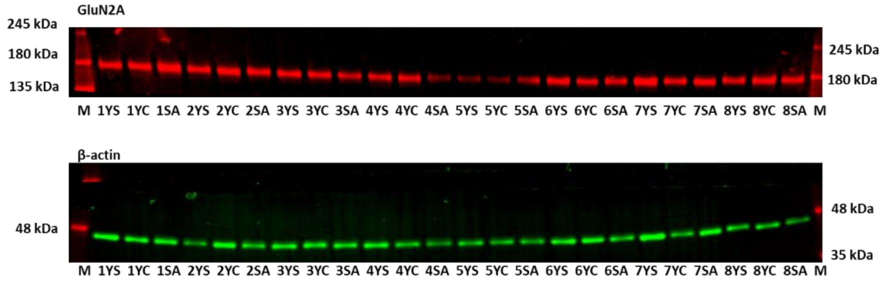
</td></tr>
</table>

GluN2B

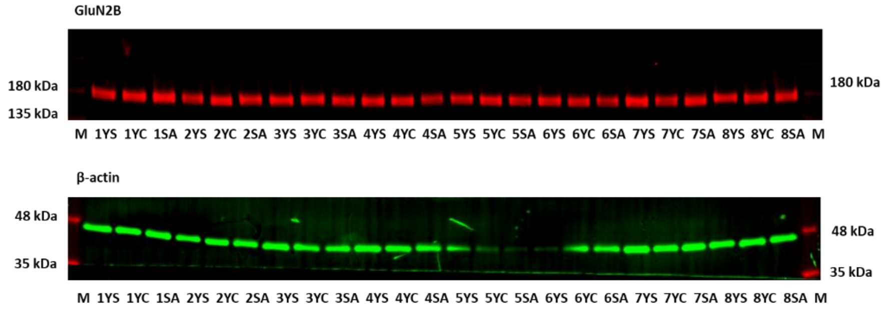

Cocaine abstinence without instrumental task

GluN1

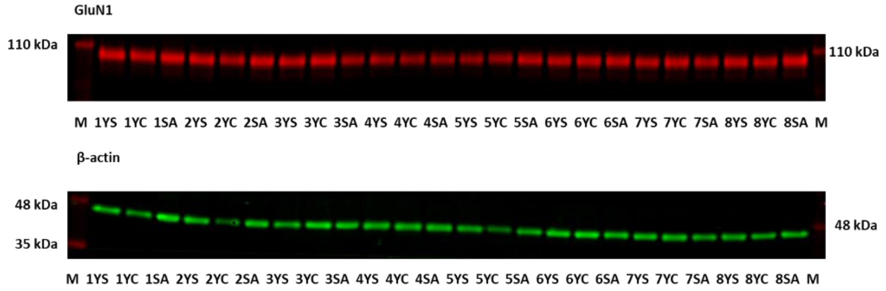

GluN2A

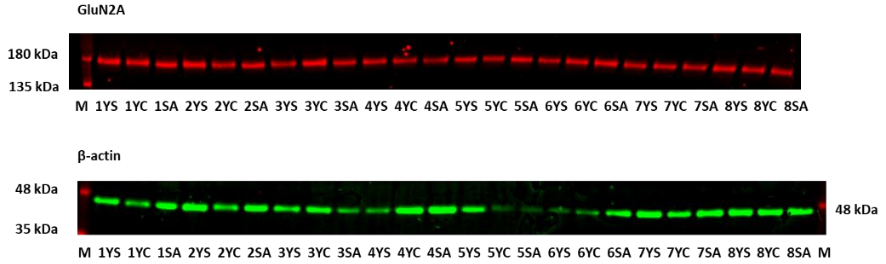

GluN2B

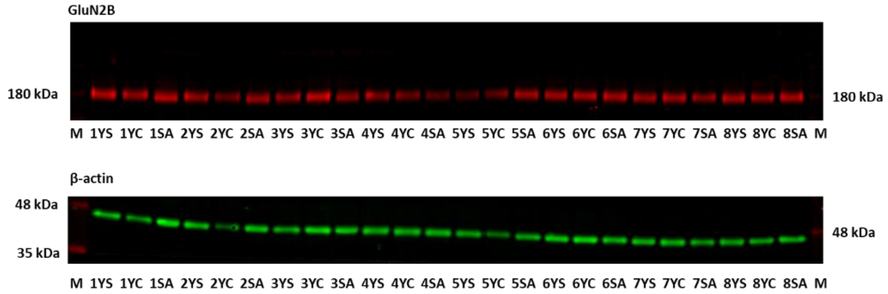

Supplement: Supplementary file 1 [file molecules-25-03480-s001.pdf]
